# Noninvasive Positive Airway Pressure Treatment in Children Less Than 12 Months of Age

**DOI:** 10.1155/2016/7654631

**Published:** 2016-06-15

**Authors:** Adetayo Adeleye, Alice Ho, Alberto Nettel-Aguirre, Jeffrey Buchhalter, Valerie Kirk

**Affiliations:** ^1^Division of Respiratory Medicine, Alberta Children's Hospital, 2888 Shaganappi Trail NW, Calgary, AB, Canada T3B 6A8; ^2^Division of Neurology, Alberta Children's Hospital, 2888 Shaganappi Trail NW, Calgary, AB, Canada T3B 6A8; ^3^Department of Pediatrics and Community Health Sciences, University of Calgary, Alberta Children's Hospital Research Institute for Child and Maternal Health, 2888 Shaganappi Trail NW, Calgary, AB, Canada T3B 6A8

## Abstract

*Study Objectives*. We identified the associated conditions of patients less than 12 months of age who were referred for polysomnogram (PSG) studies. We collated PSG findings and physician interpretation. We determined the correlation between the recommended treatment by the PSG interpreting physician and actual prescribed treatment by the referring or subjects' physician. We determined adherence with noninvasive positive airway pressure (PAP) treatment.* Methods*. This was a retrospective cohort study. Participants included children less than 12 months of age referred for PSG studies between 2007 and 2012.* Results*. 92 patients under the age of 12 months were included in the study analysis. Mean (standard deviation, SD) age in days at time of the PSG study was 208.5 (101.2). 35 (38%) patients had a diagnosis of Trisomy 21. Seven (8%) patients had no prior diagnosis. Median (Q1, Q3) apnea hypopnea index (AHI) was 22.5 (11.3–37.0). Agreement between the PSG interpreting physician's recommendation and actual prescribed treatment by the referring or subjects' physician was 85.9% [95% CI 77.1–91.6]. Mean (SD) percentage days with PAP therapy usage more than 4 hours was 25.2% (32).* Conclusions*. In our experience, despite consistent physician messaging to families, adherence with noninvasive PAP treatment is low.

## 1. Introduction

Sleep related breathing disorder (SRBD) represents a spectrum of conditions ranging from habitual snoring to frank obstructive and/or central sleep apnea that occurs in all age groups including infancy. With the relatively recent establishment of the medical specialty of Paediatric Sleep Medicine, obstructive sleep apnea (OSA), in particular, has gained much attention. OSA has been hypothesized to be a dynamic process that occurs when muscles of the upper airway collapse during inspiration coupled with other factors such as sleep state, pressure flow airway mechanics, and respiratory drive [1–3].

A recent comprehensive review of the clinical utility of PSG in children by the American Academy of Sleep Medicine identified limited published data on infants less than 12 months of age with suspected SRBD or related conditions [4]. Patients in this age group that may be referred for PSG studies include children with craniofacial abnormalities, others with genetic conditions that predispose to sleep related breathing disorders, and otherwise well infants with symptoms of obstructive sleep apnea on historical reporting [5]. Clinically, our impression is that when SRBD is identified by PSG in this age group, there are obstacles to provision of optimal treatment such as failure to tolerate noninvasive positive airway pressure (PAP) and/or oxygen therapy, suboptimal mask fit, and inconsistent messaging to parents from multiple health care providers.

Given the demand for PSG studies in our pediatric population in general, the relatively high cost of the study, and the definite inconvenience of laboratory study to families, we sought to evaluate our practice and confirm that results from PSG testing in this age group would result in clinical intervention. We also describe our experience with PAP treatment in this patient group.

The purpose of this retrospective cohort study was to identify firstly the associated conditions of patients less than 12 months of age who were referred for PSG at the Alberta Children's Hospital (ACH), which is a tertiary pediatric hospital, during a 5-year period from 2007 to 2012, inclusive. Secondly we wanted to document PSG findings and physician interpretation. Thirdly we sought to determine the agreement between recommended treatment by the PSG interpreting physician and actual prescribed treatment by the referring or subjects' physician. Lastly we wanted to appraise our experience with PAP therapy and document adherence with prescribed treatment in this age group.

## 2. Methods

### 2.1. Data Collection

Charts of infants meeting study inclusion criteria referred for PSG at the ACH from 2007 to 2012, inclusive, were reviewed. All children less than 1 year of age (corrected gestational age for premature infants) at the time of their PSG were included in the study. There were no exclusion criteria.

Data extraction templates created in a RedCap database were generated to collect information including patient characteristics (age, gender), age at time of referral, diagnosed genetic conditions, other associated medical conditions, date of PSG, interpretation of PSG, recommended treatment by PSG interpreting physician, actual prescribed treatment, and adherence with recommended treatment.

A priori determined treatment groups included no treatment recommended, PAP, supplemental oxygen therapy, combination of supplemental oxygen therapy and PAP, clinical correlate, and other. Clinical correlate was used to define situations when treatment decision was deferred to the patient's referring physician, or when more historical details were needed to recommend the most appropriate treatment. In most cases this meant an additional follow-up clinical appointment with the patient's family. Patients in the clinical correlate group could also subsequently have been prescribed PAP or supplemental oxygen as clinically indicated. Other represented patients that did not fit in the above described categories.

Data was collected from the Sleep Laboratory records and database as well as hospital charts. Adherence data for PAP therapy was obtained from parental reporting, sleep staff PAP coordinator charting notes, and computerized download data from PAP treatment units prior to the patients follow-up PSG. Specifically, we recorded percentage of days PAP therapy was used for more than four hours.

### 2.2. Polysomnography Evaluation

Computerized laboratory PSG was performed according to the Paediatric American Academy of Sleep Medicine (AASM) guidelines [6]. Monitoring included electroencephalogram (F4-M1; C4-M1; O2-M1, F3-M2; C3-M2; O1-M2 of the 10–20 international system for electrode placement), electrooculogram (right and left outer canthi), submental and bilateral anterior tibialis surface electromyograms, 3-lead electrocardiogram, thoracic and abdominal excursion (Respitrace plethysmography), oronasal airflow (thermocouples and nasal pressure), end-tidal carbon dioxide monitoring, transcutaneous carbon dioxide monitoring, and finger oximetry.

Sleep architecture and staging was determined using standard electroencephalogram (EEG) criteria. Obstructive apnea was defined as a reduction in airflow of ≥90% associated with continued abdominal and chest wall motion lasting 2-3 breaths in duration. Hypopnea was defined as a peak signal excursion drop by ≥50% of preevent baseline lasting 2-3 breaths in duration associated with EEG arousal and/or ≥3% drop in oxygen saturation. Central apnea was defined as a reduction in airflow of ≥90% associated with no evidence of abdominal or chest wall motion lasting 20 seconds in duration or lasting for 2-3 breaths and associated with an arousal or a ≥3% drop in oxygen saturation. Lastly, obstructive sleep apnea was defined as an overall apnea hypopnea index of greater than 1.5 per hour of total sleep time.

### 2.3. Data/Statistical Analysis

This was a retrospective descriptive cohort study. Descriptive statistics (mean, median, and standard deviation, and interquartile [IQ] 25th and 75th values, frequency histogram) was used for analysis and reporting of results. 95% confidence intervals (alpha significance level = 0.05) are presented. We characterized the PSG findings of the group as a whole using means (SD) if the data had a normal or at least symmetric distribution and medians (IQR) if distribution was not normally distributed or skewed. For the subgroup of patients with SRBD, observed on PSG, we identified the specific treatment recommendations made by the interpreting physician (i.e., PAP, supplemental oxygen, or a combination of both, no treatment or clinical correlate). We also recorded the actual treatment ordered by the subject's responsible/referring physician in order to determine the agreement between the recommended and actual prescribed treatment. In addition to those subjects who have been treated with PAP therapy, we described adherence patterns. The current definition of adequate adherence with PAP is not standardized but is generally based on percentage days with usage more than 4 hours. This parameter was recorded at the 6-month mark following when the PSG was performed or based on information available prior to the patients follow-up PSG.

## 3. Results

A total of 92 patients under 1 year of age met our inclusion criteria. Demographic and descriptive polysomnogram data for these subjects is shown in Table 1. There were 54 (58.7%) boys.

The most common occurring comorbidity was Trisomy 21 (35/92 patients). Seven (7.6%) patients had no associated comorbidity at the time of referral for PSG. The group classified as “other” included 16 patients with 15 unique diagnoses (CHARGE syndrome occurring twice). In addition to their unique diagnoses, 2 patients also had a diagnosis of laryngomalacia (Table 2).

Table 3 shows the different predetermined a priori treatment options and the distribution of patients to each treatment group. Agreement between the recommended (i.e., dictating/interpreting PSG physician recommendation) and actual prescribed treatment (i.e., treating/referring physician action) was 85.9% 95% CI (77.1, 91.6).

In actuality, a total of 49 patients were prescribed PAP therapy (i.e., treatment by the patient's referring physician). Six of the patients in the clinical correlate group were prescribed PAP treatment. A total of 17 patients were prescribed supplemental oxygen. Four patients from the clinical correlate group received supplemental oxygen treatment. Of the 49 patients who were prescribed PAP therapy, 20 patients had objective PAP compliance data downloaded from their PAP therapy machine. The mean (SD) percentage days with usage greater than 4 hours was 25.2 (32). Of the remaining 29 patients without objective PAP therapy download information, we documented evidence of some attempt to use PAP therapy from parental reporting (*n* = 15) or PAP coordinator notes (*n* = 13).

## 4. Discussion

The identified comorbidities in our patient cohort are similar to previously reported findings in patients referred for PSG. Leonardis and colleagues also identified Trisomy 21 as the most common genetic syndrome in a cohort of 126 infants who had OSA diagnosed by PSG at less than 12 months of age [5]. Given the associated comorbidities we identified in our study, it is not surprising that these patients are referred for formal overnight PSG studies. Most of the patient comorbid conditions are associated with high morbidity and mortality. This is supported by the high median AHI identified in this patient cohort. Assessment of gas exchange also showed that cumulative average oxygen saturation was less than 90% for 12% of the total recorded sleep time. Rhein and colleagues recently published normative values in a cohort of term and preterm infants not requiring supplemental oxygen. They reported an average (SD) cumulative time less than 90% saturation of 3.6% (3.5) [7].

We observed an 85.9% (95% CI 77.1–91.6) agreement between the recommended treatment by the interpreting PSG physician and the actual treatment prescribed by the referring physician. We were encouraged to identify consistent messaging from physicians to families as we had assumed the poor compliance with PAP therapy in this age group might be related to prescribing physician behaviour.

Our data confirmed that many of the infants referred for PSG at this young age had what would generally be considered clinically significant SRBD, requiring treatment. Just over half (53%) of referred patients were prescribed PAP treatment. Seventeen patients (18.5%) were prescribed supplemental oxygen therapy. Three patients were prescribed both supplemental oxygen and PAP therapy (3.3%).

We also identified adherence patterns in patients who had been prescribed PAP therapy. The parameter we recorded was percentage days with usage more than 4 hours. Of the 49 patients prescribed PAP therapy, only 20 (41.7%) patients had objective download information in our sleep staff PAP coordinator charts. The average percentage adherence with PAP therapy defined as percentage days with usage more than 4 hours was only 25.2% in the 20 patients with objective PAP download data. It should be noted that there are no well-defined objective definitions of PAP adherence, which makes direct comparisons difficult [8]. Marcus et al. report a mean nightly PAP use of 5.3 (2.5) hours in children aged 2 to 16 years over a 6-month study period determined by objective download CPAP information [9]. A study from our own center in children of similar age group reporting objective download data over a 46-month follow-up period showed a mean daily PAP use of 4.7 hours and overall adherence with on-going CPAP use of 67% [10]. Several investigators have described low adherence with PAP treatment as well as parental overestimation of actual PAP usage [9]. Predictors of PAP adherence continue to be actively investigated. DiFeo et al. recently reported that PAP adherence in children and adolescents is related primarily to family and demographic factors rather than severity of apnea or measures of psychosocial functioning [11]. In our study, often treatment could not be adhered with due to ineffective PAP mask interface device. This can be a problem in small infants, those with T21, and infants with craniofacial abnormalities.

Limitations of this study include its retrospective design, the small number of patients with objective PAP adherence data, and the fact that our findings are not generalizable to a healthy population of infants. Access to PSG testing is limited at our center due to budgetary constraints; thus we have developed specific referral requirements. Only physicians in the Departments of Paediatric Otolaryngology, Respirology or Sleep may submit requisitions for PSG testing. In general, we are not able to accommodate requests for PSG in children with adenotonsillar hypertrophy or other surgical airway issues associated with symptoms of SRBD unless they are considered high risk for surgery [4]. If symptoms persist following other medical and/or surgical management approaches, a PSG is performed in order to confirm ongoing clinically significant SRBD and to titrate PAP. The population described in this cohort include infants in whom no alternative surgical therapy is considered appropriate and PSG is performed to identify optimal PAP and/or oxygen therapy. In addition the decision of treatment with supplemental oxygen was often at the discretion of the interpreting PSG physician. In such cases oxygen therapy would also have been trialed in the laboratory to ensure it supported respiratory pattern and that there was no ensuing hypoventilation. Hence our referred population for PSG particularly in the less-than-1-year age group is a highly biased cohort of patients. The referral process at our centre also introduces another potential bias as children with the most severe disease may have been referred directly for surgical intervention and not for PSG.

It is hard to generalize why some patients were successful with PAP therapy initiation, while others were not. A qualitative interview and/or questionnaire analysis of the patient families' experience with initiation and compliance with treatment is now being considered as a routine tool in our laboratory.

This study provides information on the practicality of performing PSG in this patient population. In spite of the labour and resource intensive process it provides additional useful clinical information in regard to the severity of SRBD that may be present. This study, however, also shows that despite the severity of SRBD, patients often have difficulty being established on PAP therapy. This calls for more thought into how studies are performed in this age group. Perhaps a modified study which ensures normal gas exchange for age may be sufficient until the child and family is at a point where effective treatment is more likely to be successful. This is especially noteworthy given that obstructive sleep apnea in infants has a distinctive pathophysiology, natural history, and treatment compared with that of older children and adults as discussed by Katz et al. in their recent publication. Infants have both intrinsic anatomical and physiological predispositions toward airway obstruction and gas exchange abnormalities [12]. For example, the increased chest wall compliance and ventilator control instability that are present at birth naturally improve with age. It is interesting to note that in the study by Leonardis and colleagues assessing infants who had OSA diagnosed by PSG at less than 12 months of age, the most common treatment intervention was treatment of reflux disease (68%) followed by observation (26%) [5].

In summary, our study showed that our cohort of patients had severe SRBD. Treatment was frequently indicated for the SRBD. Recommended treatment by the PSG interpreting physician and the actual treatment prescribed by the referring physician showed high agreement suggesting consistent messaging from physicians. Adherence with treatment however was low. The utility of PSG in infants less than one year of age needs to be further and systematically evaluated by a prospective study in order to confirm that the additional information provided by testing has an overall positive impact on infants and their families.

## Figures and Tables

**Table 1:** Patient demographic and descriptive polysomnogram data.

	Mean (SD)	Median (IQ values)
Age (days) at time of polysomnogram study	208.5 (101.2)	186.0 (130.2–277.0)

AHI	33.2 (36.6)	22.5 (11.3–37.0)

Minimum SpO_2_ (%) TST	80.1 (8.5)	82.5 (77.4–85.6)

Mean SpO_2_ (%) TST	94.1 (3.1)	94.9 (92.8–96.0)

% TST SpO_2_ < 90%	11.9 (22.1)	1.3 (0.3–8.0)

% TST SpO_2_ < 80%	1.4 (8.2)	0.0 (0.0–0.1)

Mean PETC0_2_	44.8 (7.6)	43.0 (40.8–46.9)

% TST mean PETCO_2_ > 50 mmHg	14.0 (27.3)	0.0 (0.0–10.3)

AHI: apnea hypopnea index; TST: total sleep time; PETCO_2_: end tidal carbon dioxide; SpO_2_: oxyhemoglobin saturation by pulse oximetry.

**Table 2:** Frequency of associated conditions.

Associated condition	*N* (%)
Trisomy 21	35 (38.0)
Prematurity^~^	9 (9.8)
Pierre Robin syndrome	9 (9.8)
Pulmonary hypertension	3 (3.2)
Achondroplasia	6 (6.5)
Thoracic dystrophy	1 (1.1)
Prader-Willi	2 (2.2)
ALTE^#^	2 (2.2)
Genetic other	2 (2.2)
Other^*∗*^	16 (17.4)
No associated comorbidity	7 (7.6)
Total	92

^*∗*^((1) laryngomalacia/micrognathia/microcephaly. (2) Infantile spasms. (3) Hypotonia NYD. (4) Congenital myotonic dystrophy. (5) CHARGE syndrome. (6) Freeman Sheldon syndrome. (7) Hypotonia. (8) SMA Type 1. (9) Cutis marmorata telangiectasia congenital. (10) Potocki-Lupski syndrome. (11) CHARGE syndrome. (12). Agenesis of corpus callosum. (13) Macroglossia. (14) Benign epilepsy of infancy. (15) Pulmonary stenosis/VSD. (16) Diastrophic dysplasia/laryngomalacia).

^~^Two patients with prematurity also had a documented diagnosis of chronic lung disease.

^#^Apparent life-threatening event (ALTE).

**Table 3:** Treatment groups showing dictating PSG physician recommendation versus treating/referring physician action. The percentages represent agreement within each category.

Treating/referring physician action	Dictating PSG physician recommendation					
	No treatment^A^ *N* (%)	Oxygen *N* (%)	PAP *N* (%)	Oxygen + PAP *N* (%)	Clinical correlate *N* (%)	Other *N* (%)
No treatment^B^ *N* (%)	9 (75%)	0 (0%)	3 (7.1%)	0 (0%)	4 (16%)	1 (50%)

Oxygen *N* (%)	1 (8.3%)	8 (100%)	1 (2.4%)	0 (0%)	0 (0%)	0 (0%)

PAP *N* (%)	1 (8.3%)	0 (0%)	38 (90.5%)	0 (0%)	0 (0%)	1 (50%)

Oxygen + PAP *N* (%)	0 (0%)	0 (0%)	0 (0%)	3 (100%)	0 (0%)	0 (0%)

Clinical correlate *N* (%)	0 (0%)	0 (0%)	0 (0%)	0 (0%)	21 (84%)	0 (0%)

Other *N* (%)	1 (8.33%)	0 (0%)	0 (0%)	0 (0%)	0 (0%)	0 (0%)

^A^ Table 3 when read in the vertical direction shows the dictating PSG physician recommendations. The no treatment column indicates that of the total 12 patients who were recommended no treatment, 9 patients (75%) received no treatment, 1 patient received oxygen, 1 patient received PAP, and 1 patient received other therapy.

^B^The table read in the horizontal direction indicates the treating/referring physician's action. For the no treatment group, 9 patients received no treatment (75%) correlating with what was recommended. No patients in the oxygen group ended in the no treatment group. Three patients in the PAP group received no treatment. No patients in the oxygen + PAP group ended in the no treatment. Four patients in the clinical correlate group received no treatment and 1 patient in the other group received no treatment.
